# Supplementary material for: Implementation of Guidelines for Healthier Canteens in Dutch Secondary Schools: A Process Evaluation
Source: Int J Environ Res Public Health. 2019 Nov 15;16(22):4509. doi: 10.3390/ijerph16224509 (PMC6887932; doi:10.3390/ijerph16224509)
Supplement: Supplementary file 1 [file ijerph-16-04509-s001.pdf]

International Journal of
Environmental Research and Public Health

Implementation of Guidelines for Healthier Canteens in Dutch Secondary Schools: A Process Evaluation

Irma J. Evenhuis [1,*], Ellis L. Vyth [1], Lydian Veldhuis [2], Suzanne M. Jacobs [2], Jacob C. Seidell [1] and Carry M. Renders [1]

Supplementary Materials

Table S1. The stated questions to assess the perceived factors affecting implementation, including the Cronbach's Alpha.

Factors	Items	Questions	Answer Options	Baseline	After Intervention	Conclusion
Individual factors						
Knowledge	3 items	1. Role Clarity: For me it is clear what activities I have to do to make the school canteen healthier.<br>2. Knowledge: I have all the information I need to make the school canteen healthier.<br>3. Knowledge: I have enough knowledge to make the school canteen healthier.	5-point Scale	<0.60	<0.70	Analyse separate
Self-efficacy	6 items	1. I find it easy to do my tasks regarding the implementation of a healthier school canteen.<br>2. Other tasks conflict to perform my tasks for the healthier school canteen.<br>3. I am confident that I can change the offer in the canteen.<br>4. I am confident that I can increase the number of healthier products in the canteen.<br>5. I am confident that I can change the accessibility of healthier products of the canteen.<br>6. I am confident that I can perform my tasks, even if there are barriers (e.g., lack of time/motivation of colleagues)	5-point Scale	>0.70	>0.70	Analyse together
Attitude–Belief and Outcome expectations	9 items	*See below*	5-point Scale	>0.70	>0.70	Analyse together
Attitude-Beliefs	3 items	1. I find it pleasant to do my tasks regarding the implementation of a healthier school canteen.<br>2. I feel good, when I perform tasks regarding the healthier canteen.<br>3. I feel myself stressed/sad/nervous, when I	5-point Scale	>0.70	<0.60	Analyse together with outcome expectations

		perform tasks regarding the healthier canteen.				
Attitude-Outcome expectations	6 items	1. The effects of a healthier school canteen are visible for me.<br>2. Performing my tasks regarding a healthier school canteen gives me many advantages.<br>3. If I perform my tasks for the healthier school canteen the canteen will get a healthier offer.<br>4. If I perform my tasks for the healthier school canteen the accessibility of a healthier offer in the canteen will increase.<br>5. If I perform my tasks for the healthier school canteen students will eat more healthily.<br>6. Performing my tasks for the healthier school canteen, gives me satisfaction.	5-point Scale	>0.70	>0.60	Analyse together with beliefs
Social Influence	3 items	1. Descriptive Norm: Colleagues perform their healthier school canteen activities good.<br>2. Subjective Norm: Other people expect me to perform my healthier school canteen activities good.<br>3. Social Support: I am receiving enough support in performing my healthier school canteen activities	5-point Scale	<0.60	<0.70	Analyse separately
Routine	2 items	1. Regularly, I control whether I perform all my tasks for the healthier school canteen.<br>2. For me it is easy to remember what I have to do to create a healthier school canteen.	5-point Scale	>0.70	>0.70	Analyse together
Intention	1 item	I want to create a healthier school canteen in the coming 6 months.	5-point Scale	X	X	X
Motivation	1 item	I am motivated to create a healthier school canteen.	5-point Scale	X	X	X
Skills	1 item	My skills are sufficient to create a healthier school canteen.	5-point Scale	X	X	x
Professional Role	2 items	1. In my opinion, it is my task to change the offer of food and drinks in the canteen, to a healthier offer.<br>2. In my opinion, it is my task to change the accessibility of the canteen, to a healthier accessibility.	5-point Scale	>0.70	>0.70	Analyse combined
Behavioural regulation	3 items	1. Self-monitoring: Performing my tasks for the healthier school canteen has become a habit.<br>2. Action Planning: I am having a clear plan of how I will perform my tasks for the healthier school canteen properly.<br>3. Action Planning: I am having a clear plan of how I will perform my tasks for the healthier school canteen properly, even	5-point scale	>0.70	>0.70	Analyse together

		when there will arise barriers (lack of time, unmotivated stakeholders).				
Environmental factors						
Need for support	3 items	1. I need more information to perform my tasks regarding a healthier school canteen.<br>2. I need more training to perform my tasks regarding a healthier school canteen.<br>3. I need more support to perform my tasks regarding a healthier school canteen.	5-point scale	>0.60	>0.70	Analyse together
Innovation	2 items	1. The guidelines are in consistence with my usual work.<br>2. The guidelines can be adapted to the vision of my school.	5-point scale	<0.60	<0.60	Analyse separately
Perceived organisational support	7 items	1. There are sufficient financial resources to perform my tasks regarding the healthier school canteen.<br>2. I perceive having enough time to perform my tasks regarding the healthier school canteen.<br>3. In my organisation, the coordination of the healthier school canteen is well arranged.<br>4. In my organisation, we made formal agreements about the tasks regarding the healthier school canteen.<br>5. In my organisation, we have enough human resources to perform the tasks regarding the healthier school canteen.<br>6. In my organisation, we have enough facilities to perform the tasks regarding the healthier school canteen.<br>7. In my organisation, I am being informed about the process of the healthier school canteen regularly.	5-point scale	>0.70	>0.60	Analyse together

Table S2. The stated questions to assess the quality of implementation, including the Cronbach's Alpha.

Factors	Items	Questions	Answer Options	After Intervention	Conclusion
Dose received	1 item	Have you received the [implementation tool]	Yes/No	X	X
Dose received	1 item	Have you read/used the [implementation tool]	Yes/No	X	X
Satisfaction with:					
Schools' questionnaire	1 item	This questionnaire was understandable.	5-point Likert Scale	X	X
Stakeholders questionnaire	1 item	This questionnaire was understandable.	5-point Likert Scale	X	X
Canteen Scan	3 items	1. It is clear how the Canteen Scan needs to be filled out.	5-point Likert Scale	>0.70	Analyse together
		2. The given advices are applicable to our situation.			
		3. It is feasible to fill out the Canteen Scan.			
Advisory meeting and report	3 items	1. The advisory report was clear.	5-point Likert Scale	>0.70	Analyse together
		2. The advisory report gives practical advices.			
		3. The advisory report gives feasible advices.			
Communication materials (information brochures, poster, website-banner)	6 items	1. The given information and advices in the information brochure were clear.	5-point Likert Scale	>0.70	Analyse together
		2. The given information was practical applicable.			
		3. The given information was feasible.			
		4. The brochure 'Guidelines for Healthier Canteens' was clear.			
		5. The brochure 'Guidelines for Healthier Canteens' gives practical information.			
		6. The information and advices in the brochure 'Guidelines for Healthier Canteens' are feasible.			
Online community	5 items	1. It was easy to post messages on the Facebook community.	5-point Likert Scale	>0.70	Analyse together
		2. It was clear how to use the Facebook community.			
		3. The Facebook community gives practical applicable information.			
		4. The Facebook community gives feasible information.			
		5. I will advise colleagues to make use of the Facebook community.			
Newsletter	6 items	1. The information in the newsletter was clear.	5-point Likert Scale	>0.70	Analyse together
		2. The newsletter contained practical information.			
		3. The newsletter contained feasible information.			
		4. I was satisfied with the length of the newsletter.			
		5. I was satisfied with the attractiveness of the format.			

		6. I was satisfied with the frequency (once per 6 weeks) of the newsletter.			
Student's fact sheet	5 items	1. The fact sheet was clear.	5-point Likert Scale	>0.70	Analyse together
		2. The fact sheet was practical and applicable to our situation.			
		3. The fact sheet gives feasible information.			
		4. I was satisfied with the length of the fact sheet.			
		5. I was satisfied with the attractiveness of the format.			
